# Cortical auditory response and language development in children with microcephaly by Zika virus

**DOI:** 10.1016/j.bjorl.2026.101770

**Published:** 2026-06-16

**Authors:** Ana Cláudia Figueiredo Frizzo, Kelly Cristina Lira de Andrade, Isabela Tiezi Rombola, Klinger Vagner Teixeira da Costa, Eduardo Federighi Baisi Chagas, Pedro de Lemos Menezes

**Affiliations:** a Universidade Estadual de São Paulo (UNESP), Faculdade de Filosofia e Ciências, Department of Speech Therapy and Postgraduate Program in Speech Therapy, Marilia, SP, Brazil; b Universidade Federal de Alagoas, Biotechnology in Health ‒ RENORBIO, Maceió, AL, Brazil; c Universidade Estadual de Ciências da Saúde de Alagoas (UNCISAL), Maceió, AL, Brazil; d Universidade Estadual de São Paulo (UNESP), Faculdade de Filosofia e Ciências, Marília, SP, Brazil; e CESMAC School of Medicine, Maceió, AL, Brazil; f Universidade Estadual de São Paulo (UNESP), Rio Claro, SP, Brazil; g Universidade de Marília (UNIMAR), Marília, SP, Brazil; h Universidade de Marília (UNIMAR), Interdisciplinary Master's Program in Structural and Functional Interactions in the Rehabilitation, Marília, SP, Brazil; i CESMAC Health Research Graduate Program, Maceió, AL, Brazil

**Keywords:** Auditory development, Zika virus, Microcephaly, Auditory evoked potentials

## Abstract

**Objective:**

This study proposes to assess the auditory and linguistic development of children with microcephaly due to maternal Zika virus infection and to understand the influence of this condition on auditory cortical responses and language development.

**Methods:**

The cross-sectional descriptive study was carried out in the northeast of Brazil. Twenty children (mean age 27-months), of both sexes, with and without microcephaly due to Zika virus participated in the study. For auditory cortical responses, 40 natural /ba/ and /da/ monoaural speech sounds were presented at 70 dB HLeP. The Early Language Milestone Scale assessed the development status of auditory and visual functions related to language. For statistical analysis, covariance measures (ANCOVA) and Pearson's correlation tests were used. The Bonferroni post-hoc test was applied to significant F-values (p < 0.05). The Smart tools EP software was used to improve the investigation of the electrical response and to bring accurate and automated analyzes.

**Results:**

Longer values were observed in the latency of the N1-P2 components and a higher N1 amplitude of the cortical responses in children with microcephaly. Changes in the scale showed a significant impairment in language development in these children. Smart showed abnormalities in the neurotransmission of acoustic characteristics related to frequency information in the auditory pathway in children with microcephaly.

**Conclusion:**

The measurements of the study showed a pattern of atypical development of the auditory and linguistic system in children with microcephaly due to the Zika virus.

**Level of Evidence:**

3*.

## Highlights

• A virus that recently emerged as the Zika virus.
• Zika virus cause such as neurological, auditory changes.
• Can lead to speech and language problems and cognitive deficits.
• The CAEP enable the study of the auditory pathway’s neurofunction.

## Introduction

Brazil has faced an epidemic of the Zika virus since the beginning of 2015 when the first suspected cases with microcephaly were confirmed in the states in the northeast of Brazil.^1^ The transmission of the virus occurred through contaminated amniotic fluid in contact with the fetus during pregnancy, which caused severe pre, perinatal and postnatal neurological damage.^2^, ^3^, ^4^

The impact of the Zika virus on the development of children with microcephaly is still little known. It is known that the pattern of neurological disorders varies according to the age of the maternal infection and that changes in central functions are expected in most cases, which significantly compromises neuropsychomotor development, vision and hearing.^5^

Some auditory changes do not correlate exclusively with the ability to hear, but also to decode and process acoustic information accurately, as sound travels a long way to reach the auditory cortex.^6^ Effective listening skills and integrity are fundamental to language acquisition and development.

Several studies highlight the need to employ a model for assessing perceptual processing skills, allowing the analysis of precursors of language development through objective data, especially electrophysiological tests. The Brainstem Auditory Evoked Potential (BAEP) and the Cortical Auditory Evoked Potential (CAEP), when used together, enable the study of the auditory pathway’s neurofunction from the most peripheral regions to the cortical areas.

On the other hand, the Early Language Milestone Scale provides parameters for behavioral observation and investigates the status of the development of auditory and visual functions related to language and has been used in children with impaired global development.^7^

Until recently, few studies have investigated cortical auditory responses in children with microcephaly associated with congenital Zika virus infection, especially those that relate these responses to developmental scales. Recent studies,^8^^,^^9^ analyzed Cortical Auditory Evoked Potentials (CAEP) in children with microcephaly related to congenital Zika virus syndrome. One of them reported the presence of the P1-N1-P2 complex even in children with microcephaly, preserving partial preservation of auditory cortical function,^9^ while the other reported subcortical and cortical alterations associated with communicative impairments.^8^ The diagnostic criteria currently used in the care of patients with microcephaly due to Zika virus are still being developed. Thus, this study aims to evaluate the auditory and linguistic development of children with microcephaly due to maternal Zika virus infection and to better understand the influence of this condition on auditory cortical responses and language development.

## Method

A quantitative observational cross-sectional study. Children with suspected Zika congenital syndrome participated in the study.

The inclusion and exclusion criteria are described as follows: (1) Age between 6 to 38 months; (2) Minimum auditory response at 30 dB HL; (3) Presence of microcephaly identified by clinical examination and neuroimaging with neurological changes typical of Zika virus: calcifications, brain stones and ventricular enlargement, with mother’s clinical history compatible with Zika virus and with negative results for toxoplasmosis, syphilis and cytomegalovirus serology, which characterizes a probable Zika virus syndrome.^10^ Children in the control group were selected by convenience, in the same age and sex range, invited to participate in the study through contact with daycare centers and schools in the region. To reduce the effect of confounding variables, groups were matched by chronological age and sex. In addition, all children with microcephaly included in the study had a mean head circumference of 28.0 cm, and their mean chronological age at the time of testing was 27.2-months. According to the criteria established by the Brazilian Ministry of Health^11^ during the Zika virus epidemic, microcephaly is defined as a head circumference below 32 cm at birth for full-term infants, considering sex and gestational age.

### Cortical Auditory Evoked Potential (CAEP)

The Auditory Evoked Potentials (PEA) tests were performed with the Biologic Navigator Pro equipment. The procedure was recorded using four disposable electrodes positioned after cleaning the skin in vertex-Cz (with a jumper between the channels) in reference to the right and left lobes (A2 and A1), using the two recording channels of the equipment, the ground electrode was positioned in Middle-Frontal-Fpz. The impedance was maintained at a level below 5 K ohms.

In addition, some strategies in relation to the procedure of auditory cortical potentials were essential for the completion of the exam and the data collection success in children with microcephaly and their adverse conditions. The use of a child's silent film was used to maintain alertness^12^ and also ended up promoting greater control of the child's eye and body movements.

For the CAEP recording, different stimuli were used with different acoustic contrasts in monosyllables /ba/ and /da/. Forty monoaural stimuli were randomly presented in the proportion of 50% for each stimulus /ba/ and /da/, repeated in 2 scans (minimum interval of 1 min between scans), recorded in a 500 ms window with bandpass filtering 0.1–30 Hz, 50,000× amplification, alternating polarity and 0.7 s stimulation rate.

The procedure application time was approximately 1 h. The natural speech stimuli were fluid female voices with a 180 ms duration recorded at 70 dB HLeP, extracted from the second syllable during the emission [baba], for example, in which the formants F1, F2 and F3 were obtained in their initial and stable portion. Such stimuli were developed in the Laboratory and recorded in Praat® (Version 4.2.31), in 48 KHz and 16 bits, later recorded in wave format for the insertion of the stimulus in the equipment's software.

Two scans of each stimulus were performed in order to choose the best record for identifying the wave peaks and obtaining the final result with the best quality of the children's responses. In this record, the identification of the P1-N1-P2 complex was carried out ‒ first waves that appear in the sequence and present positive - negative - positive polarity, respectively. The records obtained were analyzed by the main researcher and discussed among members of the research group experts in the field of electrophysiology to verify the agreement of the analyzes. At least two of them identified the same peak, which corresponded to 75% agreement.

The identification and marking of the peaks of the auditory responses was performed by evaluators who were blind to the group of origin of the participants, ensuring the impartiality of the analysis.

### Early language milestone scale

The Early Language Milestone Scale (ELM) assesses the receptive, expressive auditory and visual functions related to language. Were identified in each function. Direct testing (T) was considered; the parents' report (H) or the incidental observation (O). It was classified as “adequate performance” when the child's basic level was between 90% in all items, without fail. The baby whose score was over or equal to 75% in all items for age was classified as “Pass” on the ELM scale. The procedure application time was 30 min.

### Data analysis

After data collection, descriptive statistics were performed for data quantitative analysis. The Mean and Standard Deviation (SD) values of all measures were calculated. The Kolmogorov-Smirnov test was used to verify data normality. Responses were assessed by analyzing repeated measures of covariance (ANCOVA). The Bonferroni post-hoc test was applied to significant F-values. To analyze the effect of independent variables on dependent variables, a simple linear regression model was constructed using the Enter method (forced entry). The selection of independent variables was initially carried out by analyzing the correlation, considering only variables with a correlation value >0.50 and with statistical significance (p < 0.05) for the model. The r-adjusted or coefficient of determination of the percentage of variation how well observed outcomes are replicated by the model. The assumptions of multicollinearity and homoscedasticity were verified. Statistical analyzes were performed using the Statistical Package for the Social Sciences (SPSS), version 2017.

The analysis of electrophysiological responses, specifically for the determination of the absence of the CAEP components, and the calculation of the slope and wave area calculations, was initially implemented in Microsoft Excel (version 15.32) for MAC. The numerical data of the points that constituted the waveforms of all patients were extracted from the software “AEP to AESCII, Biologic®” and typed in an Excel® spreadsheet. The numerical information of the 512 points of the CAEP waves compiled and analyzed mathematically, divided by the time window of the test (533 ms) to obtain the “grand average” of the waves for both groups, for qualitative analysis characterizing the mean CAEP wave of the groups with and without microcephaly. From these numerical values per group, appropriate formulas were created for the calculations. In addition, the development of an independent application/software Smart Tools EP was convenient for processing these wave analysis functions of the children with microcephaly. The application/software provides accurate and automated electrophysiological responses based on rigorous criteria and geometric and statistical calculations for proper diagnosis.

### Smart tools EP

We opted for the development of the application (Smart Tools EP^13^ and BioConverter^14^) in the C++ programming language for desktop, with compilations for Windows and OS (MAC) from the development platform QT Creator 5.12 (Clang 10.0 Apple, 64 Bit), which may allow the implementation for mobile phones in the future and facilitate access to technology. The interface is friendly and intuitive and has database tools for accessing, editing and archiving data implemented with the MySQL C++ 8.0 connector, with good speed and easy information processing.

**Fig. 1:**
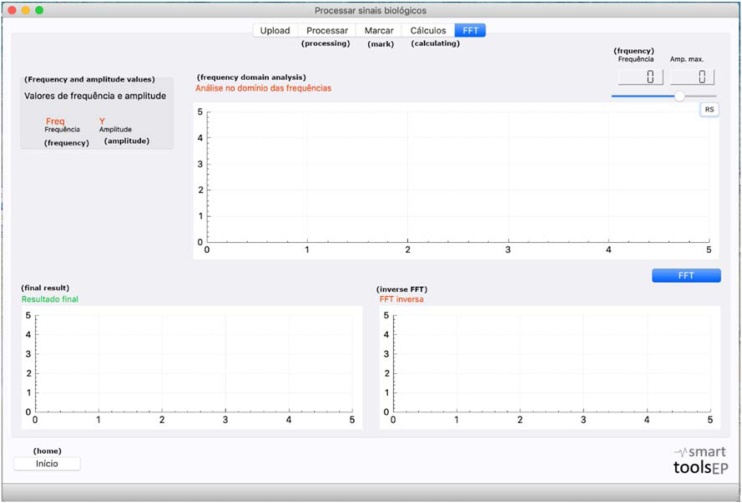
FFT analysis – software smart tolls EP.

The application/software is a support tool for applying the evoked potentials measures. The application/software presentation is currently divided into two independent programs with the following functions: (1) Test simulation; (2) Test signal processing. In this article, only the signal processing function will be used to favor the analysis of the test data. The function of the program “Process biological signals” applies the improved analysis in the response generated in the tests of auditory evoked potentials. Files can also be accessed in the program in the text version (.txt) and be processed for the execution of the most refined analysis resources. After processing, the FFT (Fast Fourier transform) calculation is performed and the analysis in the frequency domain, which determines the presence/absence of the response, expressed in a graphic (Fig. 1).

## Results

This study selected 10 children with microcephaly due to Zika virus infection and 10 control group children as a comparison group. Regarding chronological age and sex, the research group was composed of: (1) Zika group: 05 male children and 05 female children with an average age of 25-months; (2) Control group: 05 male and 05 female children with an average age of 29-months.

Table 1 shows the comparative analysis of the latency and amplitude values of the CAEP components P1, N1, P2 with the stimuli /ba/ and /da/ for the research and control groups between the right and left ears. In this comparison, the difference between the groups was confirmed, there was a significant difference between the Zika and control groups, regardless of the type of stimuli and ear: components N1 and P2 with longer latencies and higher amplitudes in the research group. There was no significant difference for the interaction or stimulus (/ba/ and /da/).

**Table 1:** Comparative Analysis OD CAEP with /ba/ e /da/ sitmulis and grupos Control and Zika.

Orelha	OD								OE										
Letra	BA				DA				BA				DA				ANOVA		
Grupo	Zika		Controle		Zika		Controle		Zika		Controle		Zika		Controle		Grupo	Letra	Interação
	Media	DP	Média	DP	Média	DP	Média	DP	Média	DP	Média	DP	Média	DP	Média	DP	p-valor	p-valor	p-valor
P1_L_IPSI	71.47	15.24	82.86	20.66	77.41	23.37	75.40	14.87	82.20	15.98	73.14	17.34	83.66	23.59	94.05	59.18	0.668	0.716	0.421
N1_L_IPSI	123.53	48.69	113.83	35.07	118.53	29.64	105.76	24.07	131.54	21.32	101.59^†^	26.18	118.95	25.07	106.71	30.29	0.051	0.899	0.515
P2_L_IPSI	179.85	51.10	159.63	31.26	178.18	34.43	159.98	38.57	195.98	33.52	149.22^†^	36.09	185.57	34.18	150.17^†^	32.02	0.002*	0.973	0.399
P1_A_IPSI	4.11	5.22	3.52	3.41	3.96	7.27	2.81	3.92	39.91	112.31	27.19	78.55	1.74	5.47	5.12	6.10	0.785	0.525	0.298
N1_A_IPSI	−1.96	3.72	0.96	5.07	−3.60	3.89	−1.18	4.28	−3.19	5.91	1.92^†^	5.27	−3.36	3.70	2.23^†^	5.82	0.013*	0.204	0.432
P2_A_IPSI	4.54	3.96	4.28	3.73	4.64	4.31	5.24	5.53	6.26	2.30	6.50	4.05	5.46	4.46	6.60	3.72	0.721	0.055	0.937
P1_L_CON	84.80	22.52	71.23	19.25	68.67	26.31	73.58	17.45	84.90	11.71	70.45	27.65	80.64	26.58	81.21	22.46	0.320	0.586	0.167
N1_L_CON	123.63	44.09	100.47	26.84	122.69	27.58	105.93	33.76	127.79	23.82	104.81	34.67	130.50	22.72	104.37^†^	24.99	0.010*	0.939	0.918
P2_L_CON	185.05	41.56	144.01^†^	23.87	178.08	25.22	162.41	23.53	196.61	39.78	162.58	42.25	201.08	42.45	156.94^†^	31.57	0.001*	0.939	0.918
P1_A_CON	3.95	5.28	3.18	4.09	3.87	6.74	2.36	4.38	3.4	5.22	30.39	92.05	3.35	5.25	4.86	5.25	0.406	0.783	0.630
N1_A_CON	−2.07	4.57	0.39	4.75	−3.57	3.44	−1.02	4.62	−2.82	5.14	0.19	6.21	−2.43	4.04	1.77^†^	4.76	0.041*	0.284	0.885
P2_A_CON	2.58	8.71	4.79	5.03	3.50	3.90	5.45	5.13	6.81	2.46	5.02	4.46	6.16	3.56	7.39	3.42	0.488	0.083	0.105

Note: Covariance Matrix Equality Test, the equality covariance matrix was met by Mix Anova test.

Comparisons between ears for the same syllable within the same group were performed using the Post-Hoc test; indicates significant difference in relation to the Zika group for the same side and syllable by the Bonferroni Post-Hoc test.

Caption: RE, Right Ear; LE, Left Ear; Syl, Syllable; M, Mean; SD, Standard Deviation; Inter, Interaction; RE-. latency (ms); A, Amplitude (μV); IPSI, Ipsilateral, CON, Contralateral.

**Indicates effect of syllable independent of the group and ear by ANOVA.

***Indicates effect of interaction between syllable and group by ANOVA independent of the ear.

* Indicates effect of the independent group of the ear and the syllable by ANOVA.

**Fig. 2:**
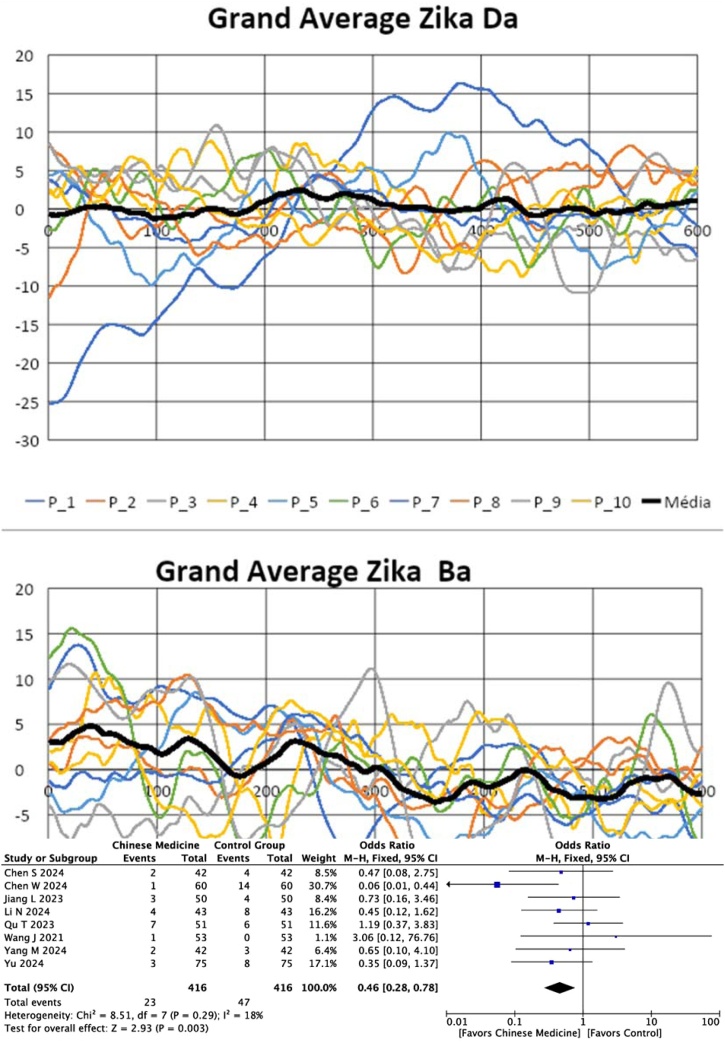
CAEP Grand average of the zika group. M, Mean.

**Fig. 3:**
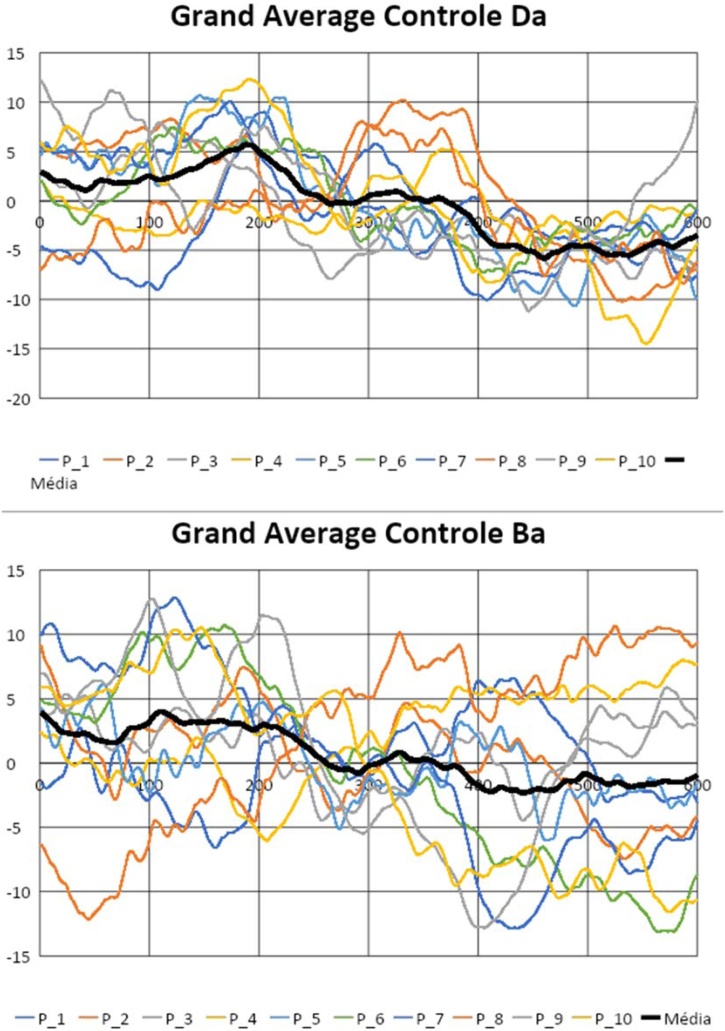
CAEP Grand average of the control groups. M, Mean.

The CAEP grand average of the two groups, control and research of the left ear with the stimuli /ba/ and /da/ are shown in Fig. 2, Fig. 3.

Regarding the ELM scale results, a very low performance was observed with failure in the receptive and expressive area of language for all items presented in the children of the Zika group. In the control group, all children performed well in items related to language and were classified as “pass”.

Table 2, Table 3 show an analysis of the correlation between the latency and amplitude of the CAEP components with the ELM Scale. A negative correlation was observed between the latency measurements of the N1-P2 neural components of children with microcephaly due to Zika virus. Low scores on the scale were associated with longer latency values and lower amplitude, regardless of stimulus and ear.

**Table 2:** Correlation Analysis of CEAP (/ba/ stimuli) with ELM Scale.

	Score ELM	
	Expressiva	Receptiva
P1_L_BA_IPSI_OE	−0.247	−0.278
N1_L_BA_IPSI_OE	−0.426^a^	−0.470^a^
P2_L_BA_IPSI_OE	−0.622^b^	−0.507^a^
P1_A_BA_IPSI_OE	−0.178	−0.165
N1_A_BA_IPSI_OE	0.524^a^	0.380
P2_A_BA_IPSI_OE	−0.033	0.046
P1_L_BA_CONTRA_OE	−0.239	−0.281
N1_L_BA_CONTRA_OE	−0.330	−0.323
P2_L_BA_CONTRA_OE	−0.428^a^	−0.368
P1_A_BA_CONTRA_OE	0.175	0.198
N1_A_BA_CONTRA_OE	0.356	0.243
P2_A_BA_CONTRA_OE	−0.250	−0.256

Values represent Pearson's correlation coeficiente.

Caption: Latency (ms); A, Ampltiude (μV).

a Indicates significant correlation by Pearson's test for p-value ≤0.05.

b Indicates significant correlation by Pearson's test for p-value ≤0.01.

**Table 3:** Correlation analysis of CAEP (/da/ stimuli) with ELM scale.

	Score ELM	
	Expressiva	Receptiva
P1_L_DA_IPSI_OE	0.143	0.144
N1_L_DA_IPSI_OE	−0.171	−0.122
P2_L_DA_IPSI_OE	−0.248	−0.476^a^
P1_A_DA_IPSI_OE	0.040	0.311
N1_A_DA_IPSI_OE	0.291	0.481^a^
P2_A_DA_IPSI_OE	0.363	0.101
P1_L_DA_CONTRA_OE	0.054	0.052
N1_L_DA_CONTRA_OE	−0.569^b^	−0.457^a^
P2_L_DA_CONTRA_OE	−0.386	−0.509^a^
P1_A_DA_CONTRA_OE	−0.117	0.156
N1_A_DA_CONTRA_OE	0.205	0.389
P2_A_DA_CONTRA_OE	0.395	0.157

Values represent Pearson's correlation coeficiente.

Caption: Latency (ms); A, Ampltiude (μV).

a Indicates significant correlation by Pearson's test for p-value ≤0.05.

b Indicates significant correlation by Pearson's test for p-value ≤0.01.

## Discussion

Regarding the CAEP measurement, the presence of the P1-N1-P2 wave complex in all children in the study, Zika and control was observed. There was 100% detectability of the neural components of the auditory system in the investigated population. Scientific evidence supports the belief that auditory cortical cyto-architecture develops early,^15^ that the presence of the P1-N1-P2 complex occurs due to the maturation of the projections of the reticular formation of the brain stem and the myelination of the thalamocortical fibers, which begins before birth and progresses to the fourth year of life.^16^^,^^17^ The identification of the neural CAEP components is compatible with efficient decoding of the syllabic linguistic information^18^ and is related to the beginning of the response to sound stimuli and the readiness for the speech sound discrimination.

Also in this sense, the use of speech sound and syllabic structure was an advantage in this study. From an acoustic point of view, with this type of stimulus, the sound energy is widely spread and has a higher concentration of medium and low frequency acoustic information, which may have been a facilitator of the response that occurs at relatively low levels of auditory processing and early maturation.^19^

On the other hand, significant differences in neurophysiological measures were observed between the Zika and control groups. The components N1 and P2 latencies were longer in the Zika group with both speech stimuli, / ba / and / da /, especially when they were presented in the left ear. In addition, the N1 amplitude was more negative in the Zika group compared to the control group, showing a difference in the pattern of development of this component in children with microcephaly due to Zika virus.

There seems to be different developmental trajectories among children in the Zika and control groups. The CAEP components N1-P2 reflect the auditory cortical activity related to the decoding of the characteristics and the perception of the acoustic changes at the beginning of the auditory neural response. In children with Zika there seems to be a failure, especially in the N1 component (evidenced by the increase in amplitude) in the control of this automatic system that monitors the perception of the change in the signal from silence to sound. The N1electrical activity is elicited essentially by the acoustic changes at the beginning of the complex sound, essential for the perception of pauses and subtle acoustic differences in speech sounds.^20^ Due to multiple neural generators, many of them late ration, changes in N1 latency values can occur until adolescence.

These findings indicate that auditory processes which precede language development can already be observed in infants.^6^ Therefore, the analysis of the P1-N1P2-N2 wave complex in young children can function as a biomarker for adverse language outcomes, especially in at-risk children.^21^

Regarding the ELM scale results, an extremely low performance was observed in the receptive and expressive area of language, compatible with delayed auditory and language development in children of the Zika group, which was associated with the negative interferences of the action of this virus on the motor and auditory sensory during the development period.^22^^,^^23^

In the comparison between electrophysiological and behavioral tests, a negative correlation was also observed between the N1-P2 neural components latency measures of children with microcephaly due to Zika virus and control group. Low scores on the scale were associated with longer latency values and lower amplitude.

There is no reference to other studies in the literature that have applied the ELM scale to children with microcephaly due to Zika virus, only with microcephaly linked to other infectious diseases,^24^^,^^25^ which also show significant language delay in children with a history of infectious diseases during pregnancy. In this study, despite the extremely low linguistic performance by children with Zika, the indicators on the scale brought parameters for comparison with electrophysiological measures, which showed the association between auditory and linguistic behavioral and neurophysiological aspects.

These results are in line with previous findings in the literature. One study observed that, although cortical detection and differentiation of speech sounds were present, they were reduced in children with microcephaly related to congenital Zika syndrome. Furthermore, they emphasized that the associations between communication performance in daily life and CAEPs highlight the value of auditory evoked potentials in the assessment of clinical populations with significant neurodevelopmental disabilities.^26^

Recent findings support the notion that children exposed to the Zika virus, even without microcephaly, may experience delays in communication development. A study with 194 normocephalic infants exposed to the Zika virus, aged between 3 and 24 months, showed that although cognitive scores remained within the typical range, their communication skills developed at a slower rate. This suggests that neurodevelopmental delays, particularly in language, may emerge later in life and reinforces the importance of early monitoring of at-risk children, even in the absence of evident brain malformations.^27^

In addition, a longitudinal case series with 15 children with Congenital Zika Syndrome (CZS), aged 14–47 months, showed that although early auditory impairments were infrequent, late-onset and fluctuating auditory changes were highly prevalent. Nearly half of the participants scored below age expectations on the Bayley III Scale in some evaluations. These findings highlight the importance of conducting auditory evaluations, including Brainstem Auditory Evoked Potentials (BAEP), in children with CZS up to at least three years of age, ensuring timely referral for treatment and language development support.^28^

The Smart tools EP was an important support tool for the more detailed assessment of auditory cortical potential measurements. The software brought relevant analysis indicators during the use of the examination signal processing function, which helped to refine the investigation of the children's central auditory function.

**Fig. 4:**
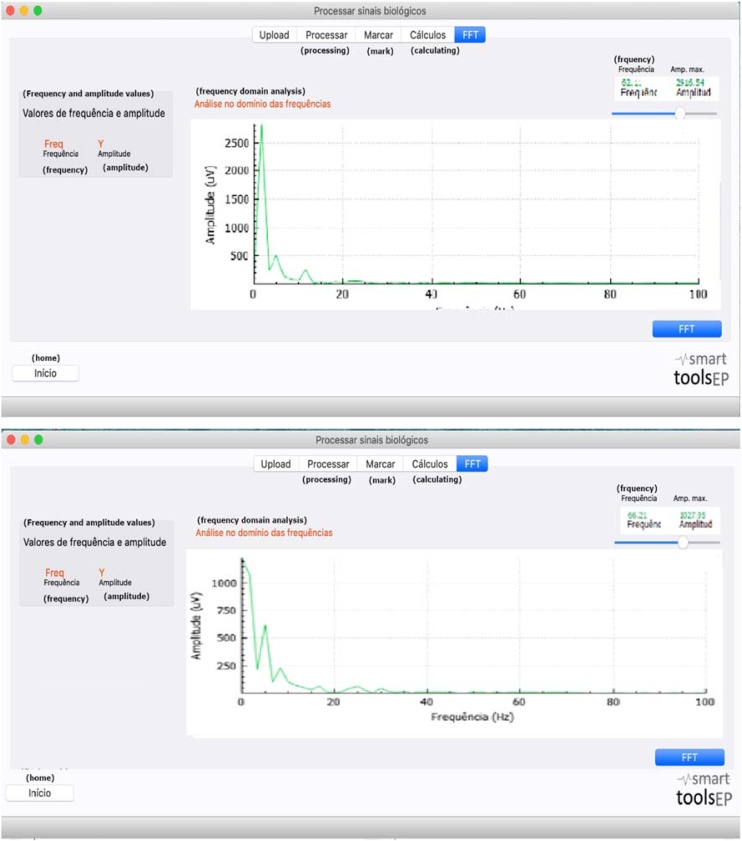
FFT Analysis of CAEP (Da stimuli) of Zika group (superior image) and control (inferior image).

First, the application of the FFT allowed to validate the presence of the electrophysiological frequency response transmitted in the auditory pathway during the CAEP test, and to analyze it in the frequency domain from the numerical information and the graphic (Fig. 4). Distinct values were observed in both groups: the Zika group showed a response with a much lower spectral characteristic and shows impairment in the neurotransmission of the acoustic information of the speech stimulus. Nevertheless, additional studies are necessary to establish normative criteria that will assist clinicians in distinguishing typical patterns, normal variants, and pathological abnormalities in future assessments.

## Conclusion

Electrophysiological and behavioral measurements showed a pattern of atypical development of the auditory and linguistic system in children with microcephaly due to Zika virus compared to children in the control group.

## ORCID ID

Isabela Tiezi Rombola: 0009-0003-1118-7181

Klinger Vagner Teixeira da Costa: 0000-0003-1425-1348

Pedro de Lemos Menezes: 0000-0003-1999-5055

## Funding

This work was carried out with the support of the Coordination for the Improvement of Higher Education Personnel – Brazil (CAPES) – Financing Code 001.

## Declaration of competing interest

The authors declare no conflicts of interest.
